# Surgical Referral and Early Diagnostic Imaging for Inguinal Hernias: A Qualitative Questionnaire Study of Australian Surgeons' Opinions

**DOI:** 10.7759/cureus.97872

**Published:** 2025-11-26

**Authors:** Adrian J Tam, Christina Kozul, Dean McKenzie, Jonathan Sivakumar, Gary Crosthwaite, Bruce Wilkie

**Affiliations:** 1 General Surgery, Eastern Health, Box Hill, AUS; 2 General Surgery, Royal Melbourne Hospital, Parkville, AUS; 3 Epidemiology and Preventive Medicine, Monash University, Melbourne, AUS; 4 Biostatistics, Epworth HealthCare, Melbourne, AUS; 5 Surgery, University of Melbourne, Melbourne, AUS; 6 General Surgery, Epworth HealthCare, Melbourne, AUS; 7 Surgery, Eastern Health, Melbourne, AUS

**Keywords:** clinical examination, family medicine/general practice, inguinal hernia, inguinal hernia repair, inguinal hernia surgery, surgery general, ultrasound anatomy

## Abstract

Introduction

Inguinal hernias are a pathology for which surgeons receive many referrals. Clinical examination remains the gold standard investigation when deciding whether an inguinal hernia repair is required. Despite this, referrals from primary caregivers will not infrequently include an ultrasound (US) or other imaging investigation. US use for the investigation of inguinal hernias in Australia is similarly on the rise. Additionally, there are no evidence-based recommendations on the imaging modality of choice when the diagnosis is less certain.

Purpose

The aim of this study was to evaluate surgeons’ experiences regarding radiological workup of inguinal hernias, particularly the prevalence of ultrasonography.

Methods

Short five-question surveys were sent out to general surgeons and sub-specialty surgeons from 2 December 2019 to 2 May 2020. Specialty surgeons included breast and endocrine, hepatobiliary, upper gastrointestinal, colorectal, paediatric, transplant, and trauma surgeons. Participation was voluntary. Data were collected using SurveyMonkey (SurveyMonkey HQ, San Mateo, CA), and data were not re-identifiable.

Results

Questionnaires were sent to 71 surgeons of different specialties. Fifty-six (78.9%) surgeons did not find US useful in hernia evaluation. Sixty-two (89.9%) would not recommend surgical intervention when clinical examination did not support US suggestive of a hernia. Despite these responses, 34 (54.8%) respondents viewed US as the most useful modality of investigation when imaging was required.

Conclusions

Over 75% of the surgeons do not recommend US in the routine workup of inguinal hernias. In cases where further investigation is required, US remains the modality of choice. However, the decision to utilize US may be better left to the operating surgeons themselves.

## Introduction

Inguinal hernias are common and represent a significant burden on healthcare resources. The estimated lifetime risk of requiring surgery for an inguinal hernia is 27-43% in males and 3-6% in females [1]. In Australia, more than 25,000 inguinal hernia repairs are performed each year [2]. Diagnostic algorithms for inguinal hernias emphasise the role of clinical examination, and radiological assessment is rarely required [3-5]. This is reflected in Safer Care Victoria's primary care medical guidelines and recommendations from the Royal Australian College of General Practitioners [6,7]. Despite this, we have anecdotally noticed that a large number of referrals for inguinal hernias are accompanied by an ultrasound (US) scan, and this corresponds with an increasing use of US in Australian practice as reported by the Australian Institute of Health and Welfare's website. Additionally, where the diagnosis is uncertain, guidelines lack high-quality, evidence-based recommendations on the imaging modality to best assess clinically 'occult' inguinal hernias.

In order to bolster the current guidelines and recommendations, the aim of this survey-based study was to evaluate Australian surgeons’ attitudes towards radiological workup of inguinal hernias, with a particular focus on the role and prevalence of US use. Our objectives were to obtain and evaluate surgeons' opinions regarding whether US was necessary in the standard pre-operative workup of inguinal hernias, how much impact US has on patients with negative clinical findings, and whether other imaging modalities had a role to play in the workup of inguinal hernias. Because inguinal hernias are routinely repaired by a range of surgeons, we sought to capture perspectives across multiple surgical subspecialties.

## Materials and methods

This qualitative observational, cross-sectional, survey-based study was conducted from 2 December 2019 to 2 May 2020. A five-question survey was created using SurveyMonkey (SurveyMonkey HQ, San Mateo, CA) and sent by email to all general and sub-specialist surgeons in Victoria via the General Surgeons Australia mailing list (Table 1). The questionnaire was self-reported, anonymous, and participation was voluntary with implied informed consent. No personally identifiable, confidential, or clinical information was collected. Data were stored securely by the primary investigator and shared only for the purposes of analysis with co-investigators.

**Table 1:** Question List Sent to Victorian Surgeons

Questions			
1. What is your surgical speciality?			
General Surgeon		☐	
Breast and Endocrine Surgeon		☐	
Upper Gastrointestinal/Hepatobiliary Surgeon		☐	
Paediatric		☐	
Trauma Surgeon		☐	
Transplant Surgeon		☐	
2. How many years have you been active as a surgeon?			
1-5 years		☐	
5-10 years		☐	
>10 years		☐	
3. Do you find ultrasound useful in the diagnosis of inguinal hernias?		Yes☐	No☐
Comments:			
4. Would you recommend a patient with symptoms, negative clinical examination, and ultrasound diagnosis of an inguinal hernia for hernia repair?		Yes☐	No☐
Comments:			
5. Of the following, which is your preferred initial imaging modality for suspected occult hernias?	US☐	MRI☐	CT☐
Comments:			

Opinions were sought from a wide range of surgeons, and respondents included general, breast and endocrine, hepatobiliary, upper gastrointestinal, colorectal, and transplant surgeons. In order to mitigate response bias, the number of questions was kept to a minimum and concise, and anonymity was ensured. Recall bias was mitigated by comparing responses to literature regarding the use of US in the workup of inguinal hernias. Surgeons were asked to identify their specialty and number of years worked, and were questioned regarding the usefulness of US, whether they would recommend an operation based on an US alone, and which modality is preferred for the workup of inguinal hernia.

Data were further stratified between general and sub-specialty surgeons to detect any difference in opinions between the two groups. An odds ratio was used, due to the small sample size, to generate a confidence interval to compare the two groups. A corresponding chi-squared test was utilized to analyse the observed frequency of responses to validate the significance of the odds ratios, with a p-value <0.05 set as significant.

This study was approved by the Research, Development, and Governance Unit of Epworth Healthcare (reference number: EH2019-456). All statistical analyses were conducted using Stata 16 (Stata Corporation, College Station, TX).

## Results

Seventy-one responses were collected from surgeons from a range of specialities and professional experience (Table 2). Fifty-six (78.9%) of surgeons stated they did not find US useful in hernia evaluation (Table 3). Additionally, 63 (89.9%) would not recommend a patient with a negative clinical examination and a positive US result for intervention. Despite these responses, 34 (54.8%) respondents still judged US to be the most useful initial investigation when imaging was required. This suggests that there exist situations where a negative clinical examination alone is not enough to rule out the need for an operation, and in these cases, US may be helpful.

**Table 2:** Respondent Specialties and Years of Experience (n=71)

Respondent Characteristics	Frequency (%)
Question 1: Primary Specialty	
General Surgeon	30 (42.3)
Breast/Endocrine	11 (15.5)
Hepatobiliary/Upper GI surgeon	15 (21.1)
Colorectal Surgeon	13 (18.3)
Paediatric Surgeon	0 (0.0)
Transplant Surgeon	2 (2.8)
Trauma Surgeon	0 (0.0)
Question 2: Years' Experience	
1-5 Years	20 (28.2)
5-10 years	13 (18.3)
>10 years	38 (53.5)

**Table 3:** Survey Results ^a^ Ultrasound vs CT (reference category). ^b^ MRI vs CT

Questions	General Surgeon (%)	Sub-specialty Surgeon (%)	OR (95% CI)	p-value (X^2^ test)	Total
Question 3: Do you find ultrasound useful in the diagnosis of inguinal hernia?					
Yes	7 (23.3)	8 (19.5)	1.26 (0.40-3.95)	0.697	15 (21.1)
No	23 (76.7)	33 (80.5)			56 (78.9)
Question 4: Would you recommend a patient with symptoms, negative clinical examination, and an ultrasound diagnosis of an inguinal hernia for hernia repair?					
Yes	5 (17.2)	2 (5.0)	3.96 (0.71-22.05)	0.116	7 (10.1)
No	24 (82.8)	38 (95.0)			62 (89.9)
Question 5: Of the following, which is your preferred initial imaging modality for suspected occult inguinal hernia?					
US	14 (58.3)	20 (52.6)	2.24 (0.67-7.55)^ a^	0.193	34 (54.8)
MRI	5 (20.8)	2 (5.3)	8.0 (1.17-54.72)^ b^	0.034	7 (11.3)
CT	5 (20.8)	16 (42.1)			21 (33.9)

## Discussion

Reliance on physical examination alone is reasonable given that sensitivity and specificity for identifying inguinal hernias are 74.5% and 96.3%, respectively [3,8,9]. In comparison, a meta‐analysis undertaken to investigate the diagnostic accuracy of US for detecting inguinal hernias found a sensitivity of 96.6% and a specificity of 84.8% [3].

The majority of comments from respondents referenced the tendency for US to be 'too sensitive' in identifying small hernias of dubious clinical significance. Another concern was the tendency of US to overinterpret spermatic cord lipoma or pre-peritoneal adipose tissue projecting into and sliding through the inguinal canal on the Valsalva manoeuvre as sonographic hernias.

**Figure 1:**
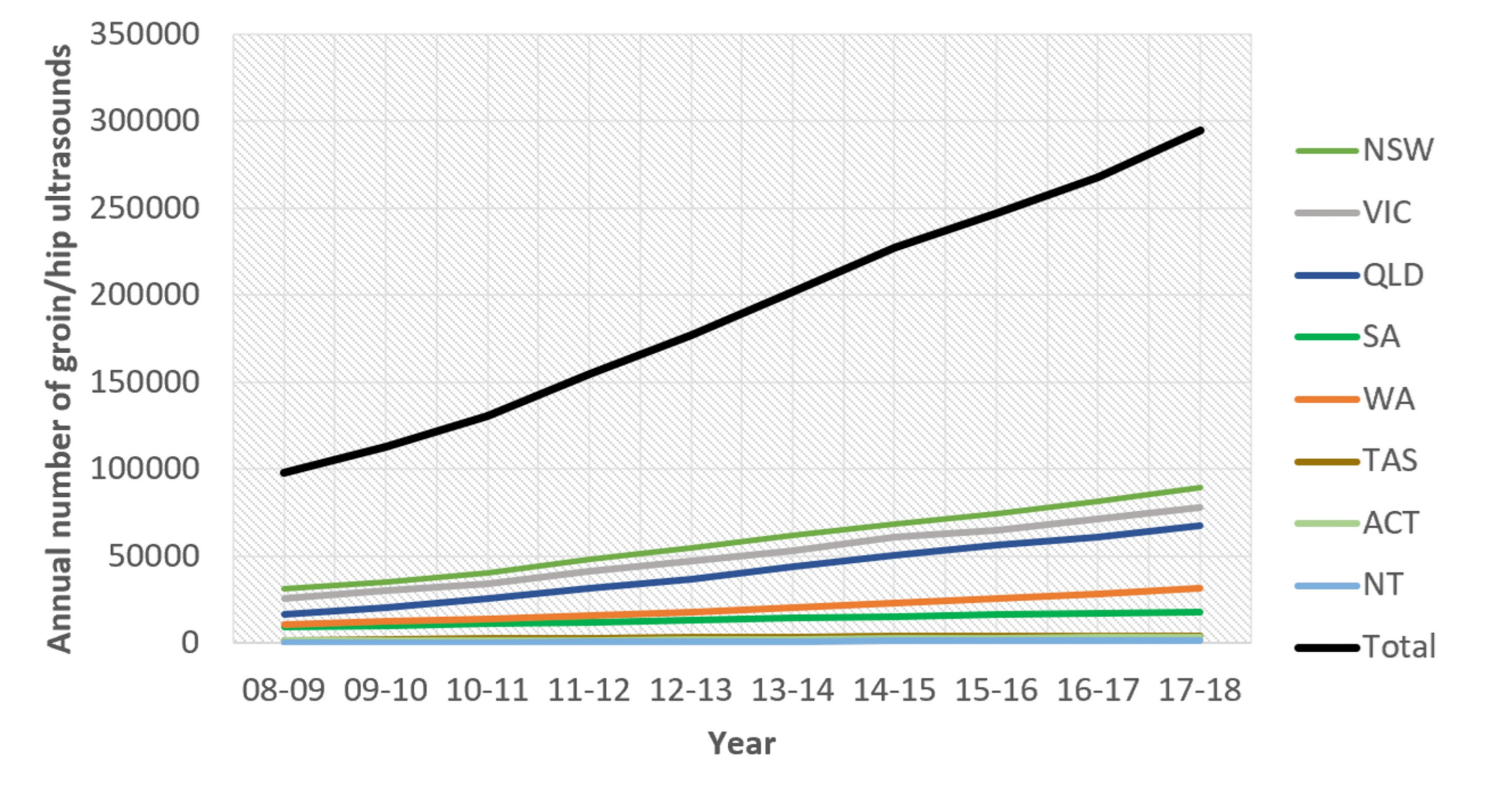
Annual Number of Australian Groin and Hip Ultrasounds From Medicare Benefits Schedule (MBS - Item Number 55816) by the Australian State (2008-2018) NSW: New South Wales; VIC: Victoria; QLD: Queensland; SA: Southern Australia; WA: Western Australia; TAS: Tasmania; ACT: Australian Capital Territory; NT: Northern Territories

While there is an increasing use of US in Australian clinical practice (Figure 1), previous reports suggest clinical examination findings have a greater influence in decision-making for surgery [3,10]. Our study reinforces current policies that US is not necessarily required in the workup of inguinal hernias, especially when clinical findings already support a decision for surgical intervention. Furthermore, a 2018 meta-analysis found that US was unreliable for the detection of clinically occult hernias [11,12]. This corresponds with the 89.9% (62 surgeons) of respondents in our study who would not recommend a surgical repair for a patient with US suggestive of an inguinal hernia, with possible symptoms, but a negative examination. Several of these respondents also suggested that symptoms (i.e. pain) in these specific patients may be unlikely to resolve with a repair and may be due to other causes. Instead, specialist clinicians advocate a period of clinical observation, with targeted investigations to rule out alternative causes. However, as a qualitative survey study, it is unknown exactly how often a patient with an inguinal hernia has symptoms due to another concurrent pathology.

Miller et al. have suggested that imaging should be considered in patients with obesity, previous surgery, radiation, trauma, or those who describe a history of intermittent pain and bulging not appreciable at the time of examination [13]. It has been proposed that US is best organised by surgeons who have an understanding of how sonographic results will impact treatment [14]. In the UK, 75% of groin hernia US requests are from surgeons in secondary care, and the remainder from family physicians [15]. This corresponds with the British Hernia Society’s recommendation that groin hernia imaging in the primary care setting is not required or recommended [16].

Overall, the findings of this study suggest that the majority of Victorian general and subspecialty surgeons agree that US is not a mandatory component of inguinal hernia workup. Although referral guidelines can differ depending on the hospital network catchment area in question, the findings of this study reinforce existing literature regarding the use of US and can help clinicians streamline referrals by avoiding delays and costs associated with ultrasonography.

Limitations

This study is limited by its questionnaire format and relatively modest sample size of 71 respondents. To mitigate expected low response rates, we expanded our study to capture perspectives from multiple surgical subspecialties. In an attempt to maximize response rates, questionnaires were limited to five questions and formatted in a way to allow surgeons to provide 'yes' or 'no' answers. To increase the depth and detail of information, respondents were given the opportunity to add comments. However, not all surgeons took advantage of this, and so some nuances in surgeons' opinions will have been missed in this way. Future research can mitigate this by capturing more responses, either with more qualitative research or with studies with larger sample sizes. Data on non-responders were not captured for practicality reasons, given the high non-response rate. In the future, this could be mitigated by utilizing multiple contact attempts and advertising the study in advance at General Surgeons Australia events. Finally, by utilizing a self-reported questionnaire, rather than capturing actual clinical practice, response and recall biases would similarly be difficult to control in a questionnaire study such as this.

## Conclusions

Over three-quarters of surgeons do not recommend US in routine work-up and diagnosis of an inguinal hernia. Instead, early specialist referral without imaging may be the most cost and resource-efficient practice. However, when imaging is required, US remains the most favoured primary imaging modality for further investigation. Clinicians referring to surgeons for consideration of inguinal hernia repair need not order US when clinical examination alone is convincing.
